# Emerging infectious diseases and the law.

**DOI:** 10.3201/eid0707.017722

**Published:** 2001

**Authors:** E P Richards

**Affiliations:** University of Missouri-Kansas City, Kansas City, Missouri 64110, USA. richardse@umkc.edu


November 1998 with the explicit mission of working to advance the elimination of trachoma and the blindness it causes. A WHO-approved strategy called SAFE is simple, sustainable, and addresses both cure and prevention:

Surgery for trichiasis—the immediate precursor to blindness
Antibiotics to treat active disease
Facial cleanliness to reduce transmission
Environmental improvement to control the agents of the disease

In ITI countries the antibiotic used is Zithromax (azithromycin), donated by Pfizer. A single oral dose of Zithromax once a year is as effective as the standard treatment of tetracycline eye ointment 2 times a day for 6 weeks. The ITI is currently working in five countries: Morocco, Tanzania, Mali, Ghana, and Vietnam. The ITI works with ministries of health to devise an operating plan and joins WHO, United Nations Children's Fund, and nongovernmental organizations to carry out this work.

**Acknowledgments**

We thank the following panelists for their outstanding contributions: Victoria McGovern, Gerald Keusch, Fabio Zicker, Natasha Martin, and Joseph Cook.

---

### Emerging Infectious Diseases and the Law

**Edward P. Richards**
University of Missouri-Kansas City, Kansas City, Missouri, USA

In the 1960s, the United States began to lose interest in public health. The development of effective vaccines and antibiotics, combined with the long-term benefits of sanitary reforms begun 100 years earlier, fostered the belief that communicable diseases had been conquered and that it was time to focus the nation's resources on chronic diseases such as cancer and heart disease. This shift led to the deterioration of the public health infrastructure, including public health law training and practice. At the same time, bioethics and the legal specialty of health law began to evolve. Both of these fields were individual-centered: bioethics concentrated in individual autonomy and health law concentrated on the delivery of, and reimbursement for, personal health services. By the 1980s, legal discourse and training on health and public health was dominated by an individual-centered jurisprudence that subordinated the public's interest to that of the individual. Although this approach resulted in important advances in patient autonomy, it undermined the public's understanding and acceptance of the traditional role of public health law—the protection of the health of the population. Many states weakened their communicable disease-reporting laws and otherwise made it more difficult to identify and manage communicable disease threats. More critically, public health professionals began to believe that they do not have the legal authority to restrict individual behavior to protect the public health and that their role is to provide personal health services on the same basis as private health care providers.

The threat of emerging infectious diseases and bioterrorism is forcing the states and the federal government to reassess the U.S. public health infrastructure and the provision of public health services, as well as to review international treaties and trade agreements to ensure that they are consistent with effective public health measures. As part of this process, it is critical to ensure that each jurisdiction has adequate legal authority to protect the health of the public and to act quickly in the face of bioterrorism or a disease outbreak. This will require the restoration of more traditional public health laws in some jurisdictions and the training of lawyers, judges, and public health professionals in public health jurisprudence. The federal government should help coordinate state efforts and should ensure that there are no federal law impediments to effective public health enforcement.

The restoration and expansion of the public health infrastructure and the development of more effective public health legal services will have many benefits beyond improving the response to emerging infectious diseases and bioterrorism. Achieving these goals is also essential to the improvement of the delivery of routine public health services such as food sanitation, immunizations, and the abatement of hazardous environmental conditions.

Dr. Richards is executive director of the Center for Public Health law at the University of Missouri-Kansas City School of Law. His research interests focus on public health and biotechnology. For more information on public health law and practice, contact the Center for Public Health Law through its Web site: http://plague.law.umkc.edu/cphl.

Address for correspondence: Edward P. Richards, Center for Public Health Law, University of Missouri-Kansas City School of Law, 5100 Rockhill Road, Kansas City, MO 64110, USA; fax: 816-235-5276; e-mail: richardse@umkc.edu

